# Retraction: A green synthesis strategy of binuclear catalyst for the C-C cross-coupling reactions in the aqueous medium: Hiyama and Suzuki–Miyaura reactions as case studies

**DOI:** 10.3389/fchem.2023.1181449

**Published:** 2023-04-04

**Authors:** Frontiers Editorial Office

The journal retracts the 29 November 2021 article cited above.

Following publication, concerns were raised regarding data misrepresentation. Specifically, patterns of repeated signals were identified in Figures 4B, 4C and 7. The authors failed to provide a satisfactory explanation during the investigation, which was conducted in accordance with Frontiers’ policies. As a result, the data and conclusions of the article have been deemed unreliable and the article has been retracted.

This retraction was approved by the Chief Editors of Frontiers in Chemistry and the Chief Executive Editor of Frontiers. The authors do not agree to this retraction.

